# A rare case of biventricular myxoma

**DOI:** 10.1186/s13019-017-0584-6

**Published:** 2017-03-27

**Authors:** Tangsakar Ermek, Naibi Aybek, Wei-min Zhang, Yong-zhong Guo, Sheng Guo, Azze Mamataly, Dong-qing Chang, Jun Liu, Zong-gang Zhang

**Affiliations:** Department of Cardiac Surgery, People’s Hospital of Xinjiang Uygur Autonomous Region, Urumqi, 830001 People’s Republic of China

**Keywords:** Case report, Cardiac myxoma, Biventricular

## Abstract

**Background:**

Cardiac myxoma is the most common primary cardiac tumor. Approximately 75–80% of myxomas are located in the left atrium. Occurrence of multiple myxomas is extremely rare.

**Case presentation:**

We describe a rare case of biventricular myxomas resulting in right ventricular inflow and tricuspid valve obstruction. The lesions were detected by echocardiography and thoracic computerized tomography (CT) and confirmed on positron emission tomography–computed tomography.

**Conclusion:**

The patient underwent successful surgical resection of the multiple cardiac myxomas. This kind of biventricular case has not been previously reported. The patient is asymptomatic as of the 10-month follow-up.

## Background

Myxoma is the most common primary cardiac benign tumor. While cardiac myxoma can occur anywhere in the heart, its etiology tends to favor a single location. Approximately 75–80% of myxomas are located in the left atrium, while 10–20% are located in the right atrium. Occurrence of multiple myxomas is extremely rare [1, 2]. In this report we present a very rare case of biventricular myxomas in which accurate clinical diagnosis permitted successful surgical excision.

## Case presentation

A 15-year-old girl was admitted to the People’s Hospital of Xinjiang Uygur Autonomous Region on July 2015 with symptoms of cardiorespiratory distress and hydrosarca. She had been complaining for 20 days of symptoms of congestive heart failure. On physical evaluation, her body temperature was 36.9 °C; respiratory rate was 24 breaths per min; blood pressure was 109/77 mmHg; and pulse rate was 113/min. She was conscious but showed a poor spirit, facial edema, pale conjunctiva and lips. On chest auscultation, thick breath sounds in both lungs and mild moist rales in both lower lung lobes were heard. On palpation, a precordial tremble and strong apex beat was noted, while listening found relative cardiac dullness and massive murmurs starting in the systolic phase and extending up to the end of diastole in the precordial area. Other findings included an enlarged liver, positive jugular vein reflux, and edema lower limbs.

**Fig. 1:**
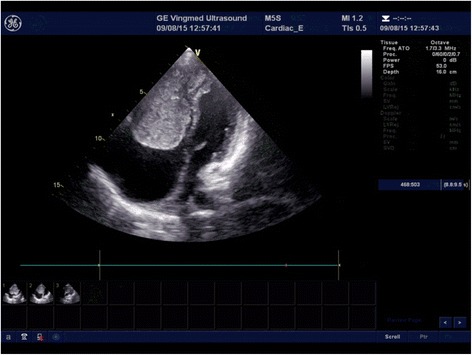
Transthoracic echocardiogram showing *right* ventricular mass

**Fig. 2:**
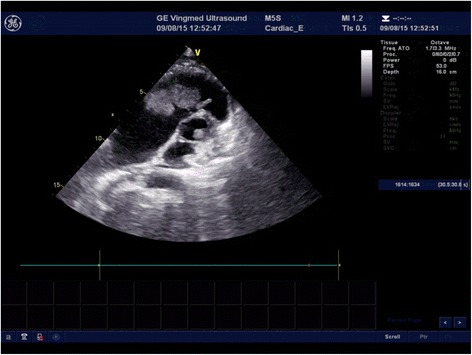
Transthoracic echocardiogram showing *right* and *left* ventricular mass

Laboratory data showed albumin hypoproteinemia (29 g/L; normal reference range 40–55 g/L) and elevated plasma levels of N-terminal fragment of brain natriuretic peptide (NT-pro-BNP: 286 pg/mL; normal level < 80 pg/mL). Serum CA-125 (tumor marker) level was elevated (164.3 ku/L; normal level, 35 ku/L) [3]. Electrocardiogram showed sinus rhythm. Brain computerized tomography was unremarkable. Thoracic radiogram showed cardiac dilatation, right lower pneumonia and pleural effusion. Transthoracic echocardiogram revealed multiple cardiac masses; a large right ventricular mass (5 cm × 4 cm) arising from the right ventricle apex was seen to prolapse through the tricuspid valve into the right atrium during systole (Fig. 1). Another small mass (1 cm × 0.8 cm) was located at the postcava near the right atrium. A third mass (1.5 cm × 1 cm) was located in the left ventricular outflow tract (Fig. 2). The left ventricle was not dilated and showed normal ejection fraction. Mild pericardial effusion was also noted.

**Fig. 3:**
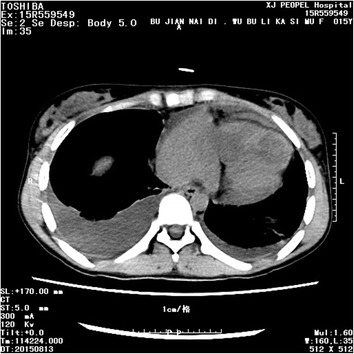
Thoracic computerized tomography image showing multiple low density shadows in the *right ventricle* and *right atrium*

**Fig. 4:**
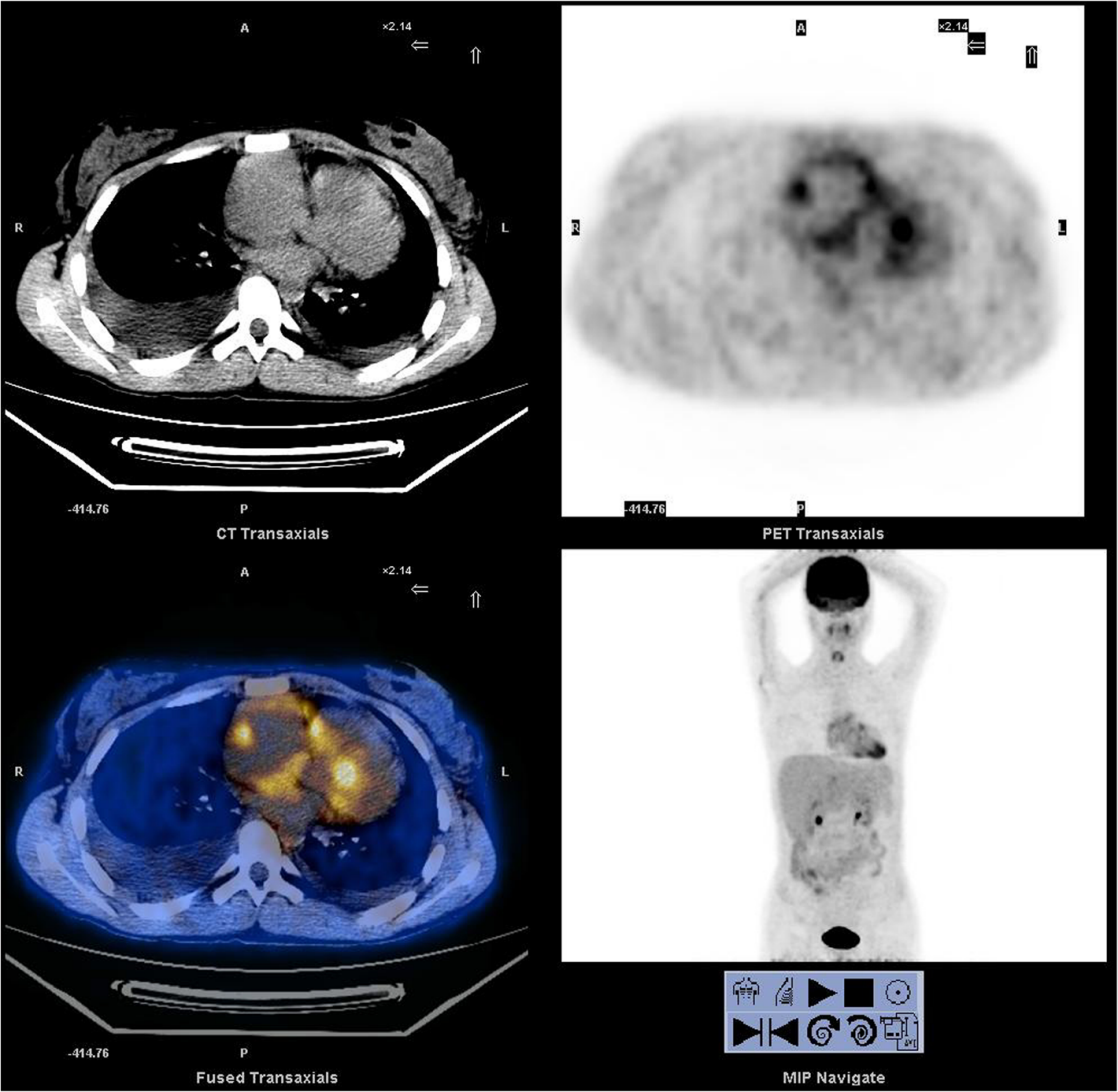
PET image showing multiple irregular masses in the *right ventricle* and *right atrium*

These findings were highly suggestive of multiple myxomas, although the diagnosis of vegetation or thrombus could not be excluded. Thoracic computerized tomography indicated a widened frontal film and pericardial effusion. Multiple low density well-delineated shadows were seen (size of the largest mass: 5.5 cm × 3.8 cm; Fig. 3). Consistent with these findings, positron emission tomography (PET) also revealed widened heart outline, especially that of the right ventricle. An irregular low-density but well-delineated shadow was observed in the right ventricle. A fluorodeoxyglucose test revealed an enhancement pattern suggestive of a benign lesion (Fig. 4), and likely a mucous tumor. No obvious malignant tumor was identified during the workup.

**Fig. 5:**
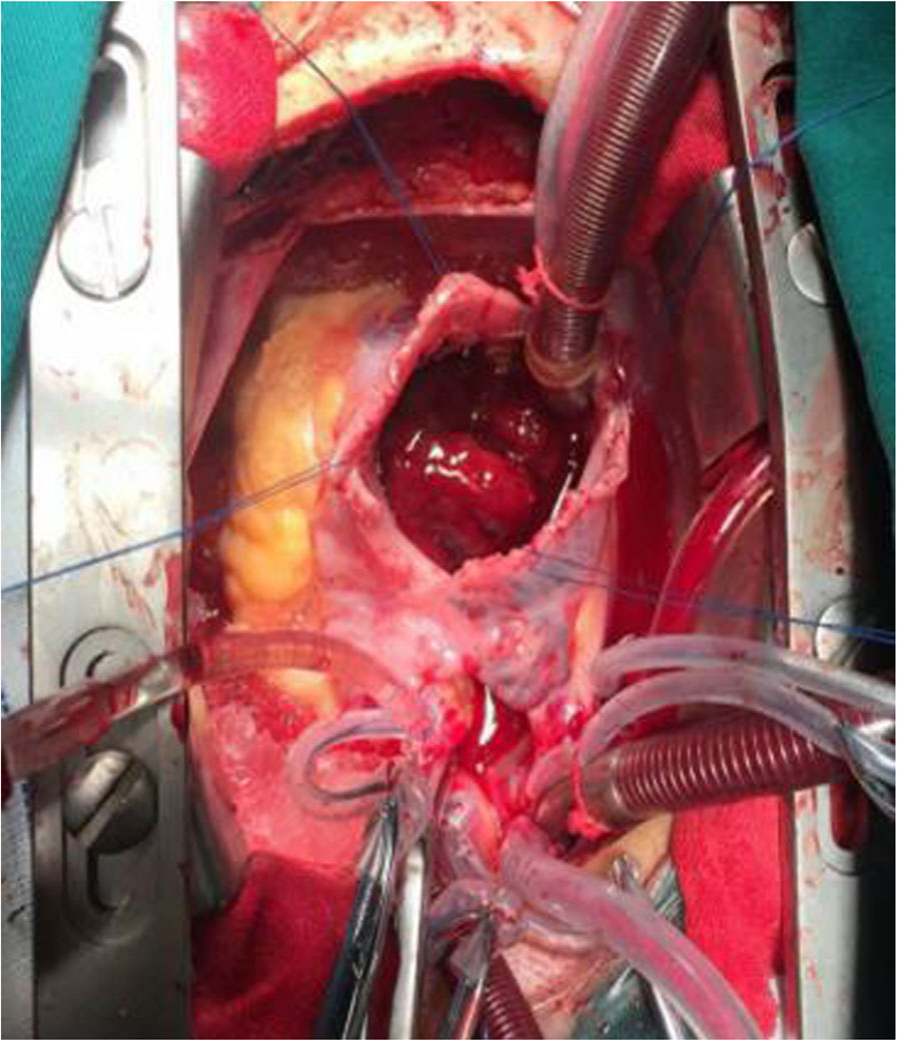
Huge tumor with thrombus in the *right* atrium detected during surgery

For about 2 weeks, the patient’s heart function and nourishment improved prior to the operation. Under general anesthesia, the chest was opened with a median sternotomy. The patient was placed on cardiopulmonary bypass; under cold blood cardioplegic arrest, the right atrium was opened widely. A massive red thrombus was seen clearly in the auricula dextra, pectinate muscles of the right atrium and postcava opening near the right atrium. A 60 mm × 45 mm red tumor was found in the right ventricle after removal of the thrombus. The tumor had prolapsed into the right atrium through the tricuspid valve (Fig. 5). Examination of the left ventricular cavity through the aortic root revealed two small pedunculated tumors (1.5 cm × 0.5 cm and 0.6 cm × 0.4 cm) with narrow pedicles arising from the left ventricular outflow tract. The tumors were excised through an aortic valve approach with a 10 mm margin. All tumors were removed successfully and the tricuspid valve was repaired.

**Fig. 6:**
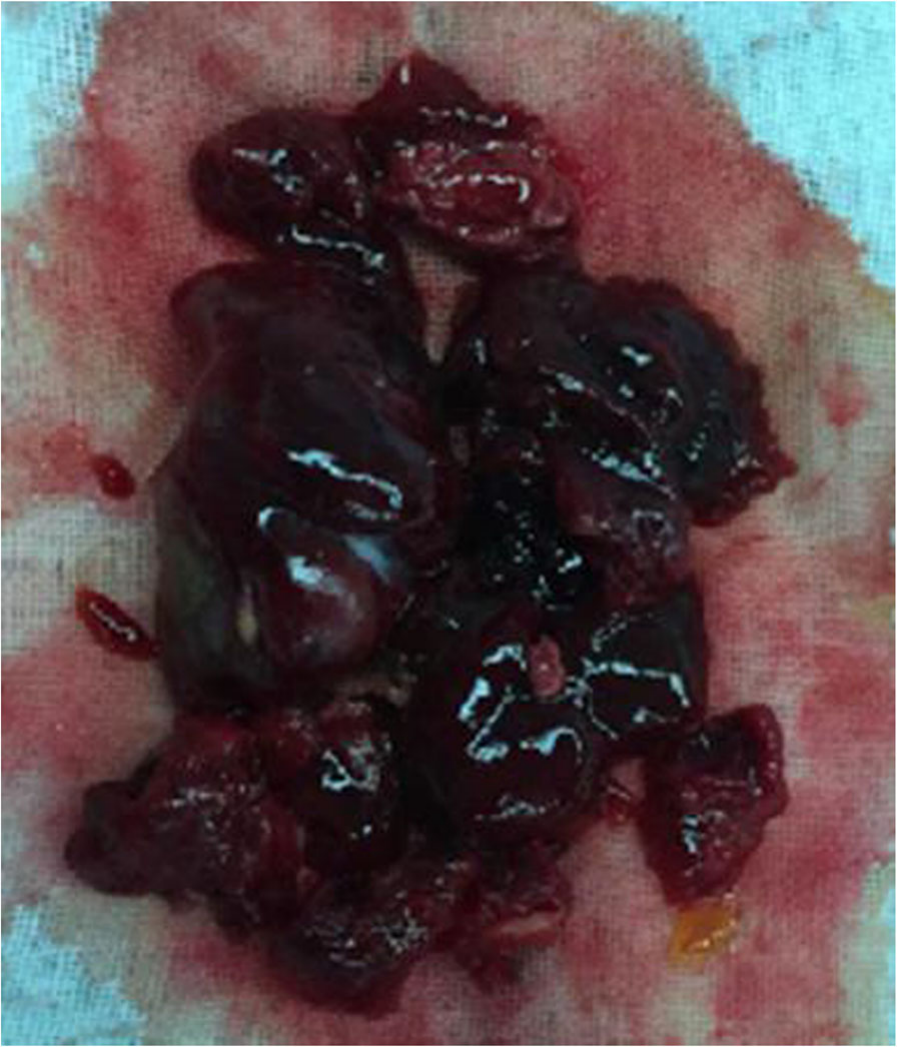
Gross appearance: a jelly-like mass with hemorrhagic areas are seen

**Fig. 7:**
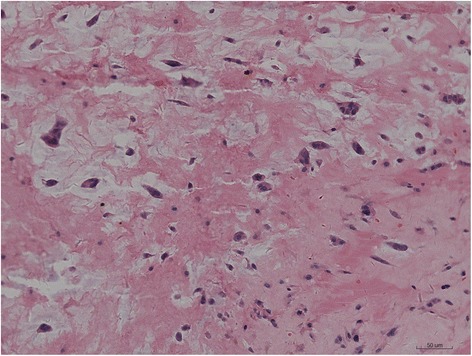
Histopathological images showing myxoid strands

The combined size of all tumors approached approximately 6.5 cm × 4.5 cm × 3.0 cm; the gross appearance was that of a jelly like mass with hemorrhagic areas (Fig. 6). Pathological examination confirmed the diagnosis of multiple cardiac myxomas (Fig. 7). During the 10 months follow-up, the patient was asymptomatic and had normal blood pressure. There were no signs of echocardiographic recurrence or valvular insufficiency.

## Discussion

Cardiac myxoma is one of the most common primary cardiac tumors, with about 75% of the tumors being benign [4]. Cardiac myxoma is more common in women, with a 2:1 female preponderance. Most commonly affected age-group is 30–60 years [5, 6]. These are rarely seen in children, and account for only 9–15% of all cardiac tumors from birth to adolescence [4]. Left atrium is the most common site of myxoma, although these may occur in the left or right ventricle.

Most myxomas have a stalk, are gelatinous, and have a broad base. The surface may be friable or villous. On histological examination, myxoid stroma can be seen stained blue in the mucus tumor cells because of the abundant mucopolysaccharide acid content. Primary cardiac tumors are classified as mucinous and non-mucinous tumors according to the histological type. Most primary cardiac tumors are mucinous tumors, while less than 5% are non-mucinous tumors.

The mucinous body tends to grow into the cardiac chamber and is connected with the wall by means of a stalk attached to the atrial septum [7]. The shape of the mucinous body can be globular, lobulated or papillary, and has a jelly-like macroscopic appearance. Most mucinous bodies feature a hemorrhagic spot and necrosis, which facilitates embolization.

Patients will often have constitutional symptoms such as anemia, fever, and weight loss. These patients may present with systemic embolization (cerebral or peripheral) or with symptoms due to intracardiac obstruction [8]. In current practice, myxomas are often discovered in asymptomatic patients, which can be easily missed and lead to delayed diagnosis. Such patients also may have a cardiac rumble, caused by obstruction of intracardiac blood flow by the tumor. Transthoracic echocardiography should be performed in all patients with suspected cardiac myxoma. In this case, the patient underwent echocardiogram, CT and PET/CT; however, the results from transthoracic echocardiography provided detailed anatomical correlates and was found to particularly valuable in preoperative risk assessment and preparation of a surgical plan.

Owing to its peculiar location, enmasse removal of the myxoma in the right ventricles is inadvisable because of the contiguity of the tumor with the cardiac muscles at the apex of the heart. Any damage to cardiac muscle, chordae tendineae, muscuil papillares, valves or conductive bundle is liable to impair cardiac function directly and may even prolong the postoperative recovery. Due diligence is required during surgery to remove multiple myxomas.

The mortality is significant between the time of diagnosis and the operative intervention because of massive embolization or total obstruction to intracardiac blood flow. Approximately, 8% of patients with myxoma die during the waiting period for surgery. The operative mortality is reported at 2–5% [9]. Therefore, after the diagnosis has been established, surgery should be performed promptly due to the possibility of embolic complications or sudden death. Because of the impaired heart function and poor general condition, the patient was treated for about 2 weeks prior to the operation. Surgical outcome is generally good; 20-year survival rate is 85% [9]. The recurrence rate after resection is approximately 5%.

The girl in the current report had multiple myxomas in the left and right ventricles, which makes it a very rare clinical case. The patient was referred for open-heart surgery and the multiple myxomas were completely removed. She recovered well after surgery; postoperative recovery and follow-up period has been uneventful till date.

## Conclusion

In conclusion, we describe our experience with successful surgical resection of multiple cardiac myxomas in a 15-year-old girl. Occurrence of biventricular myxomas has not been reported. The patient is symptomatic, as of 10-month follow-up.

## Abbreviations

CT: Computerized tomography
NT-pro-BNP: N-terminal fragment of brain natriuretic peptide
PET/CT: Computerized tomography
